# Development and validation of body fat prediction models in American adults

**DOI:** 10.1002/osp4.392

**Published:** 2020-01-15

**Authors:** Zachary Merrill, April Chambers, Rakié Cham

**Affiliations:** ^1^ Department of Bioengineering University of Pittsburgh Pittsburgh Pennsylvania; ^2^ Department of Physical Therapy University of Pittsburgh Pittsburgh Pennsylvania; ^3^ Department of Ophthalmology University of Pittsburgh Pittsburgh Pennsylvania

**Keywords:** body composition, body fat, obesity

## Abstract

**Introduction:**

Commonly used statistical models to predict body fat percentage currently rely on skinfold measures, anthropometric measures, or some combination of the two but do not account for the wide ranges of age and body mass index (BMI) present in the American adult population. The objective of this study was to develop a statistical regression model to predict in vivo body fat percentage (dual energy X‐ray) in men and women across significant age and obesity ranges.

**Methods:**

This study included 228 adults between the ages of 21 and 70, with BMI between 18.5 and 40.0 kg m^−2^. The study population was split into training (n = 163) and validation (n = 65) groups, which were used to develop and validate the prediction models. The models were developed on the training group using a backwards stepwise regression analysis, with the initial predictors including age, BMI, and several anthropometric and skinfold measurements.

**Results:**

The final statistical regression models included age, BMI, anthropometric measures, and skinfold measures with significant effects following the stepwise process. The models predicted body fat percentage in the testing group with average errors of less than 0.10% body fat in males and females, while the four previously existing methods (Durnin, Hodgdon, Jackson, and Woolcott) significantly underestimated or overestimated body fat in both genders, with errors ranging between 2% and 10%.

**Conclusions:**

The final models included hand thickness, and the female model was dependent on waist circumference and two of the skinfold measures, while the male model used hip and thigh circumferences, along with three skinfold measures. By including the skinfold measurements separately, instead of only as sums like previous models have done, these models can account for the different relative contributions of each site to total body fat.

## 1 INTRODUCTION

When considering body fat percentage (BFP) as a measurement of obesity status, high body fat, especially when combined with low body mass index (BMI), is associated with increased all‐cause mortality^1^ and cardiovascular disease mortality.^2^ While approximations of whole‐body and abdominal obesity can be approximated with measures such as BMI and waist and hip circumferences, body composition information can provide additional insight into cardiovascular health risks.^2^

While measures such as BMI, waist circumference, and waist to hip ratio can approximate BFP and related health risks, imaging methods such as dual energy X‐ray absorptiometry (DXA) scanning have been used as direct in vivo measures of body composition, including BFP.^3^ Other methods have used skinfold measurements to model BFP in the general population^4^, ^5^, ^6^ and the elderly.^3^, ^7^, ^8^, ^9^ These methods have used skinfold to directly predict BFP^3^, ^7^, ^8^ or to predict body density,^4^, ^5^, ^6^, ^9^ which can then be used to approximate BFP using the two‐compartment equation developed by Siri.^10^ The two‐compartment model developed by Siri^10^ uses assumed densities for both fat mass and fat free mass, so that BFP can be determined from the calculated body density.

Methods relying only on anthropometric measurements including circumference measurements and height^11^, ^12^, ^13^ have also been employed for the purpose of predicting BFP. These methods have the advantage of accounting for individual body shape, while not requiring the equipment and training necessary to perform skinfold measurements. Specifically, the relative fat mass (RFM) metric^13^ approximates BFP using only height and waist circumferences, making it one of the simplest methods of determining obesity status in American adults. While these established methods have proven effective for military personnel^11^, ^12^ and provided improvement over BMI alone in the American adult population,^13^ they do not account for the additional effect of age in predicting body composition. When looking at correlations between anthropometric measures such as waist circumference and BMI with body composition, previous research has found that both age and race also play significant roles in predicting BFP.^14^, ^15^, ^16^ These findings highlight how BMI alone does not provide accurate insight into body composition.^15^

Because of the wide age and obesity ranges presented in the American adult population,^17^ the known effects of age and BMI on predicting body composition,^16^ and the significant predictive abilities of skinfolds^4^, ^5^, ^6^ and anthropometric measurements^18^, ^19^, ^20^ on body composition, accurate statistical models should attempt to include all of these variables. The methods previously determined by Durnin and Womersly,^4^ Hodgdon,^11^, ^12^ Jackson and Pollock,^5^, ^6^ and Woolcott^13^ have been developed to predict BFP in Navy personnel^11^, ^12^ and the general adult population^4^, ^5^, ^6^, ^13^ with simple regression models based on easily collected measurements. Additionally, the methods utilizing skinfolds^4^, ^5^, ^6^ employ the sums of skinfolds, instead of allowing for contributions from separate skinfold sites to predict BFP individually. By contrast, this study aims to develop the most accurate body fat prediction models in American adults, with the requirement of including a larger number of input variables.

Of the reference methods observed for the purpose of comparison, the log‐skinfold method, developed by Durnin and Womersly,^4^ uses the base‐10 logarithm of a sum of four skinfolds (triceps, biceps, subscapular, and hip) to determine body density, while the sum‐skinfold method developed by Jackson and Pollock^5^, ^6^ uses a sum of seven skinfolds (chest, midaxillary, triceps, subscapular, abdominal, hip, and thigh) in its linear and quadratic terms, along with age, to determine density. The RFM method created by Woolcott^13^ avoids using skinfold measurements and only employs height and waist circumferences. The Navy method, developed by Hodgdon,^11^, ^12^ uses logarithmic terms including the abdomen and neck circumferences for men; the waist, hip, and neck circumferences for women; and height for both genders. While the log‐skinfold, sum‐skinfold, and RFM methods all use the same inputs for men and women, with differing coefficients, the Navy method is the only one that requires a different set of measurements for men and women.

The objective of this study was to develop multiple regression models to predict BFP in working men and women using all of these parameters, in order to develop a clinical tool that will provide the most accurate results, and improve over the existing prediction methods without the need for expensive imaging equipment.

## 2 MATERIALS AND METHODS

A total of 228 working adults (116 female), defined by having a full time job of at least 35 hours per week, aged 21 to 70 (mean, 44.4 ± 13.7 years; Table 1) participated in this study. Recruitment was stratified by BMI group, age group, and gender to ensure a comprehensive representation of the current working population. More specifically, male and female participants were recruited in equal numbers in three BMI categories (normal weight, 18.5 ≤ BMI < 25.0; overweight, 25.0 ≤ BMI < 30.0; and obese, 30.0 ≤ BMI < 40.0) across three age groups: young (21 ≤ age < 40), middle (40 ≤ age < 55), and old (55 ≤ age < 70). The study was approved by the University of Pittsburgh Institutional Review Board, and written informed consent was obtained prior to any data collection.

**Table 1:** Descriptive statistics for the study population

	N	Age, y	Mass, kg	Height, cm	BMI, kg m^−2^
All	228	44.4 (13.7)	81.3 (17.3)	170.0 (9.1)	28.0 (4.9)
Female	116	45.6 (13.5)	75.3 (14.4)	163.8 (5.9)	28.0 (5.0)
Male	112	43.2 (13.8)	87.5 (17.8)	176.5 (7.1)	28.0 (4.9)

*Notes*. Values are shown as mean (*SD*).

Each participant had his or her height and mass recorded in order to confirm eligibility based on BMI. Female participants of childbearing age were then required to complete a pregnancy test, with a negative result being required for eligibility. Next, nine skinfolds and thirteen anthropometric measurements were collected (Table 2). All of the arm and leg measurements were collected for the right sides only. Each measurement was collected three times, and the average of the three was used for analysis. A whole‐body DXA scan (Hologic QDR 1000/W, Bedford, MA, USA) of each participant was then collected using the same methods used in prior studies,^10^ with the participant lying supine. BFP was determined from the scan as total fat mass divided by total body mass.

**Table 2:** Anthropometric measures collected for body fat prediction

Anthropometric Variable	Definition
Chest skinfold	Diagonal fold at 45° angle: one half the distance between the anterior axillary line and the nipple for men, one third the distance between the anterior axillary line and the nipple for women
Mid‐axillary skinfold	Vertical fold, on the mid‐axillary line at the level of the xyphoid process of the sternum
Triceps skinfold	Vertical fold, on the posterior midline of the upper arm, halfway between the acromion and olecranon processes, with the arm held freely to the side of the body
Biceps skinfold	Vertical fold, on the anterior aspect of the arm over the belly of the biceps muscle, 1 cm above the level used to mark the triceps site
Subscapular skinfold	Diagonal fold, 1 to 2 cm below the inferior angle of the scapula
Vertical abdominal skinfold	Vertical fold, 2 cm to the right of the umbilicus
Horizontal abdominal skinfold	Horizontal fold, 2 cm to the right of the umbilicus
Suprailiac skinfold	Diagonal fold, in line with the natural angle of the iliac crest taken in the anterior axillary line immediately superior to the iliac crest
Thigh skinfold	Vertical fold, on the anterior midline of the thigh, midway between the proximal border of the patella and the inguinal crease (hip)
Waist circumference	Circumference at the umbilicus
Hip circumference	Around largest part of the hip
Upper thigh circumference	Around proximal thigh
Mid‐thigh circumference	Around point midway between proximal border of patella and inguinal crease
Lower thigh circumference	Around thigh 1 cm above proximal border of patella
Knee circumference	Around medial and lateral femoral epicondyles
Calf circumference	Around largest part of calf
Ankle circumference	Around medial and lateral malleoli
Upper arm circumference	Around midpoint between acromion and olecranon processes
Elbow circumference	Around medial and lateral humeral epicondyles
Lower arm circumference	Around midpoint between lateral humeral epicondyle and ulnar styloid process
Wrist circumference	Around radial and ulnar styloid processes
Hand thickness	Thickness at center of palm

Before starting the statistical analysis, the full data set of 228 participants was randomly split into two subgroups: the training set, which contained 163 participants, and the testing set, which contained the remaining 65, with each set containing similar age and BMI distributions (Table 3). The purpose of splitting the full data set into the testing and training sets is that the predictive models can be independently developed and validated on separate data sets, so that the models' performance on the testing set would be representative of the models' performance on real‐world data. Developing and validating the models on separate sets also ensure that any overfitting of the models does not occur, and demonstrate the ability of the models to predict body fat values on data that were not initially used to create the models.

**Table 3:** Descriptive statistics for the training and testing subsets

	N	Female	Age, y	BMI, kg m^−2^	BFP
All	228	117	44.4 (13.7)	28.0 (4.9)	29.3 (9.2)
Training	163	82	44.3 (13.9)	28.1 (5.1)	29.2 (9.2)
Testing	65	35	44.7 (13.2)	27.7 (4.6)	29.5 (9.1)

*Notes*. Values are shown as mean (*SD*).

All analyses were performed in JMP Pro 12 (SAS Institute, Cary, NC, USA). Specifically, a backwards stepwise regression analysis was performed on the whole‐body DXA determined BFP in the training subset within each gender group. The initial regression model contained age, BMI, age^2^, and BMI^2^ and all their interaction terms, all skinfold measurements, and circumferences of the neck, waist, hips, and limbs. In each step of the analysis, the predictor with the largest *P* value was removed, and the analysis was repeated. This process of removing the least significant predictor and repeating the analysis continued until the *P* values for all predictors were below 0.10.

Once the training set model was finalized, it was applied to the anthropometric measures in the testing set, so that the predicted and actual segment parameters could be compared in the testing set, and used as a method of validating the models. For comparison purposes, several previously validated body fat estimation methods were also applied to the testing data set. These methods included those determined by Durnin and Womersly,^4^ Hodgdon,^11^, ^12^ Jackson and Pollock,^5^, ^6^ and Woolcott.^13^ Within the testing set, the actual and predicted values were compared by calculating the absolute percent error, as well as the root mean square error (RMSE). The actual BFP values were compared with the new and existing prediction models in order to determine the average difference in predicted body fat value, ∆BFP. Within each gender, *t* tests were performed between each prediction method and the DXA measured body fat to determine if there were any significant differences, with statistical significance set at *α* = .05. Because a total of four *t* tests were performed for each gender, a Bonferroni correction was used within each gender, and a *P* value below 0.0125 was required to reject the null hypothesis.

## 3 RESULTS

**Figure 1:**
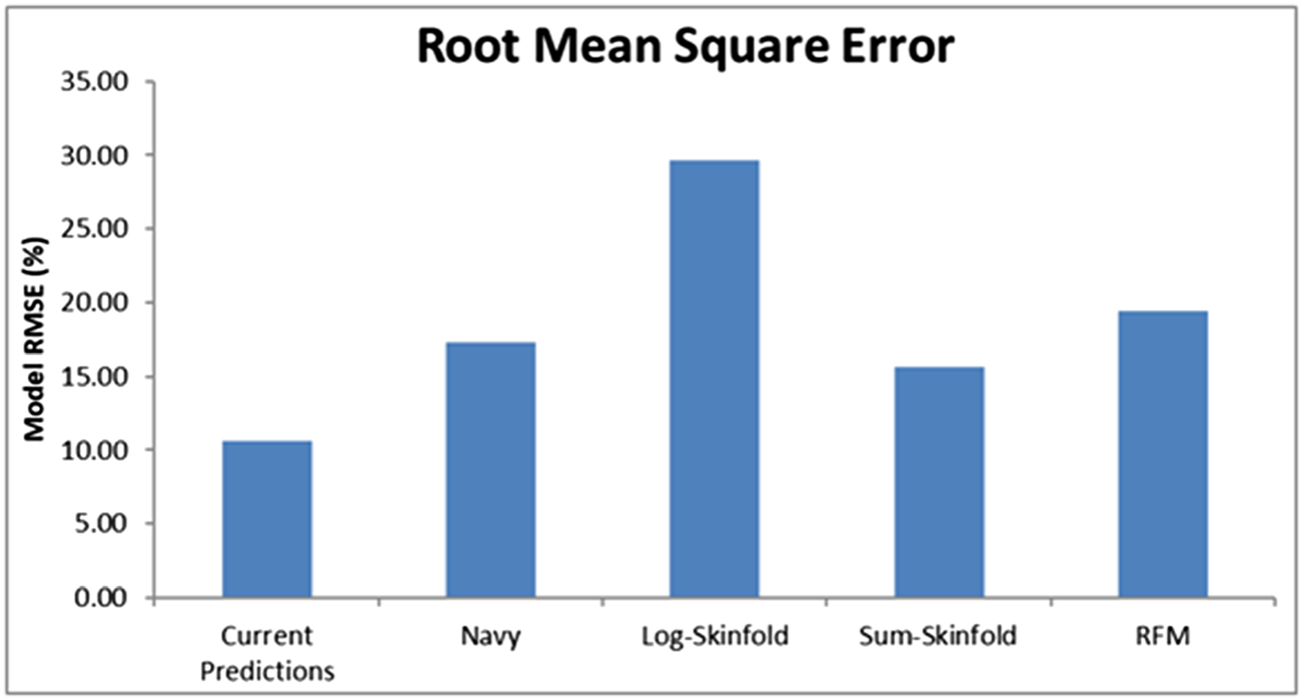
Root mean square error for the testing group for the newly developed prediction model and the Navy, log‐skinfold, sum‐skinfold, and RFM models

**Figure 2:**
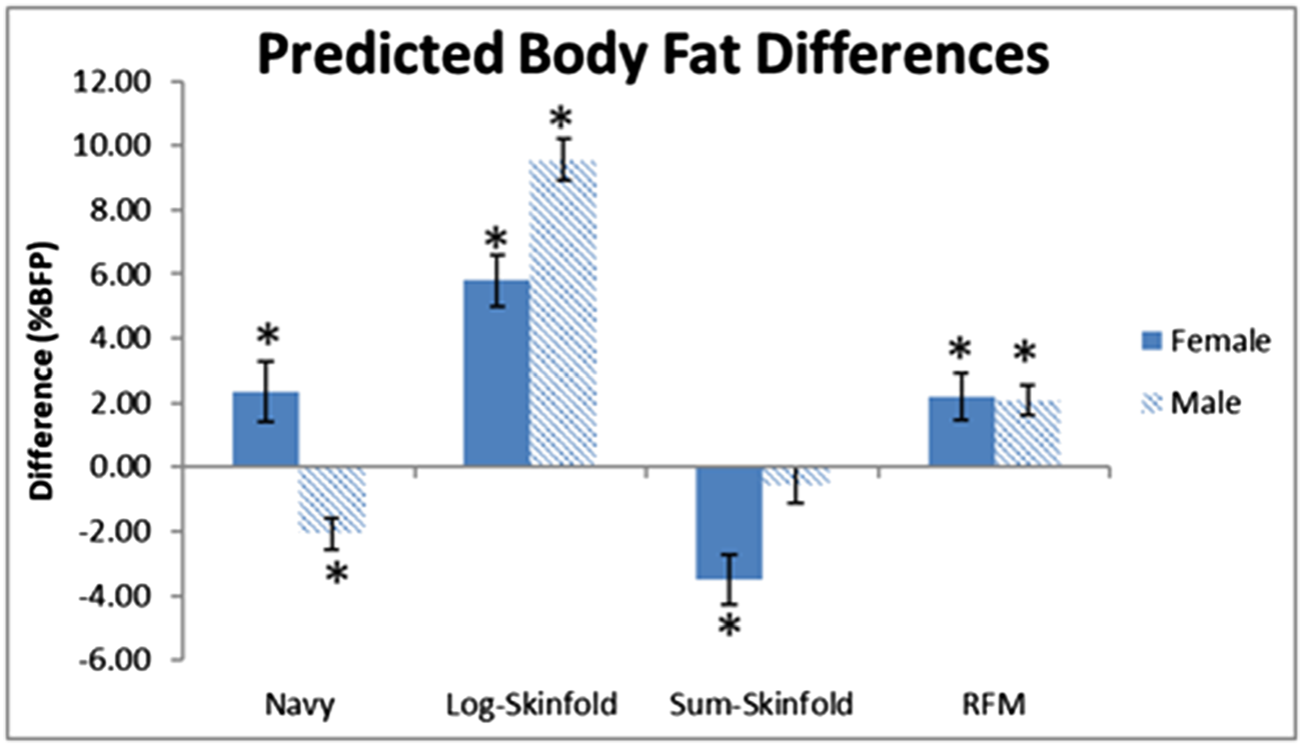
Predicted body fat differences, shown as predicted body fat minus DXA‐measured body fat for each prediction method. Error bars represent standard error. * indicates *P* < .0125 (following the Bonferroni correction) compared with DXA‐measured values

The final models predicted total BFP within the testing subgroup with an RMSE of 10.6% (Figure 1), compared with the Navy, log‐skinfold, sum‐skinfold, and RFM prediction methods, which had RMSE of 17.3%, 29.7%, 15.6%, and 19.4%, respectively. When directly comparing the body fat predictions to the DXA measured values, the log‐skinfold (*p*_male_ < 0.0001, *p*_female_ < 0.0001) and RFM (*p*_male_ = 0.0046, *p*_female_ = 0.0026) predictions significantly overestimated body fat for males and females (Figure 2). The Navy formula significantly underestimated only male body fat (*p*_male_ = 0.0034), and the sum‐skinfold method significantly underestimated only female body fat (*p*_female_ < 0.0001). Compared with the DXA measured values, the new prediction formula (Equations 1 and 2) showed a ∆BFP of less than 0.10% for both males and females (Figure 2) and average absolute errors of less than 10% (Table 4). For both equations, all circumference and skinfold measurements are measured in cm.

(1) $$\text{Female}\;\mathrm{BFP}=225.43–\mathrm{Age}*12.42+\left({\mathrm{Age}}^{2}\right)*0.140–\mathrm{BMI}*14.43+\left({\mathrm{BMI}}^{2}\right)*0.266+\left(\mathrm{Age}*\mathrm{BMI}\right)*0.893–\left(\mathrm{Age}*{\mathrm{BMI}}^{2}\right)*0.0154–\left({\mathrm{Age}}^{2}*\mathrm{BMI}\right)*0.0100+\left({\mathrm{Age}}^{2}*{\mathrm{BMI}}^{2}\right)*0.000174+\left(\text{Waist circumference}\right)*0.129–\left(\text{Hand thickness}\right)*6.44–\left(\text{Vertical abdominal skinfold}\right)*1.83+\left(\text{Thigh skinfold}\right)*1.61$$

(2) $$\text{Male}\;\mathrm{BFP}=22.76+{\mathrm{Age}}^{2}*0.00168–\mathrm{BMI}*0.700+\left({\mathrm{BMI}}^{2}\right)*0.0139–\left({\mathrm{Age}}^{2}*\mathrm{BMI}\right)*0.000233+\left({\mathrm{Age}}^{2}*{\mathrm{BMI}}^{2}\right)*0.0000072+\left(\mathrm{Hip}\;\text{circumference}\right)*0.288–\left(\mathrm{Mid}-\text{thigh circumference}\right)*0.401–\left(\text{Hand thickness}\right)*4.94+\left(\text{Subscapular skinfold}\right)*2.22+\left(\text{Vertical abdominal skinfold}\right)*1.60+\left(\text{Thigh skinfold}\right)*1.69$$

**Table 4:** Average absolute percent errors for each prediction method. Values given as mean (sd). Errors calculated as the absolute difference between prediction methods and DXA measured values, divided by DXA measured values.

	Predicted	Navy	Log‐Skinfold	Sum‐Skinfold	RFM
All	8.7 (7.6)	14.9 (11.3)	33.3 (26.0)	11.3 (8.7)	14.7 (13.8)
Female	8.4 (6.4)	13.2 (9.9)	20.3 (17.1)	12.4 (9.4)	11.2 (11.6)
Male	9.1 (8.9)	16.9 (12.7)	48.5 (26.6)	10.1 (7.8)	18.8 (15.2)

The final prediction models included several age and BMI terms for males and females and accounted for over 80% of the variation in BFP, with *R* ^2^ values of.85 and.87 for females and males, respectively. The only anthropometric measures included in the female model were waist circumference and hand thickness, while the male model included hip and mid‐thigh circumferences, and hand thickness. Both models also included the vertical abdominal and thigh skinfolds, while only the male model includes the subscapular skinfold.

## 4 DISCUSSION

The objective of this study was to develop a new prediction model for body fat estimation in working men and women, accounting for a wide range of ages and obesity levels and variation in individual anthropometry. Compared with previous methods of estimating body fat in adults, this new formula showed greatly improved accuracy in predicting the DXA‐derived BFP, as demonstrated by the RMSE, ∆BFP, and average absolute errors. The new prediction model is unique among other methods because it includes age, BMI, anthropometric measures, and skinfold measures, while the preexisting methods only use skinfolds (Durnin^4^), anthropometric measurements (Hodgdon^11^, ^12^ and Woolcott^13^), or skinfolds and age (Jackson^5^, ^6^). Because the backward stepwise regression process initially included all of the predictors, this study did not suffer from the limitation of being restricted only to specific categories of inputs.

Compared with the established methods of predicting BFP, the new model requires more measurements than the anthropometry‐only methods used by the Navy^11^, ^12^ and RFM^13^ equations, but with the benefit of providing significantly more accurate predictions in both men and women. While the RFM model provides significant improvement^13^ over BMI alone for predicting body composition, it results in estimations that are about two percentage points higher than the new models (Figure 2). The RFM^13^ study and this study both used DXA scans to determine the actual body composition in participants; however, the RFM study recruited a larger sample size of American adults, while this study was limited to working adults with full time jobs. Although physical activity information was not collected as part of this study, previous research has indicated that men and women with active full‐time jobs and men with sedentary full time jobs tend to be more active than unemployed adults,^18^ so these potential differences in lifestyles and activity levels between the two study populations may have contributed towards different modeling outcomes for predicting BFP.

A similar comparison issue arises when observing the differences between the Navy^11^, ^12^ models and the results of this study, because of the Navy models being developed for active duty US Navy personnel. The Navy studies observed participants that were both younger and with less excess body weight^11^, ^12^ than the participants in this study. Interestingly, this difference in population and models used leads to the Navy method underestimating body fat in males, while overestimating body fat in females, indicating that with the increasing age and BMI, circumference measurements may play different roles in predicting body composition, and skinfold measurements are necessary for providing further predictive ability.

When comparing the log‐skinfold^4^ and sum‐skinfold^5^, ^6^ measurement models to the results of this study, the log‐skinfold models greatly overestimated body fat in men and women, while the Jackson model underestimated body fat in women. The sum‐skinfold model for men was the only prediction in this comparison to not significantly overestimate or underestimate the DXA scan–derived BFP. While both of these studies observed wide age ranges of adults (16‐72 years for log‐skinfold and 18‐61 years for sum‐skinfold) similar to this study, the study populations both had lower average BMI and BFP, again demonstrating that the results of this study are necessary for use in the American working adult population.

Another major difference between the results of this study and previous skinfold models is that both the log‐skinfold^4^ and sum‐skinfold^5^, ^6^ models used sums of skinfolds to predict body composition instead of looking at the individual contributions of these variables, while this study observed the contributions of each skinfold site individually. Observing each site individually allows changes in each site to be weighted differently relative to each other. For example, Equation (2) (for males) shows that changes in the subscapular skinfold site lead to larger changes in body fat prediction than changes in the abdominal or thigh sites, while the log‐skinfold or sum‐skinfold models would treat similar changes in any of the included sites as having the same impact on body fat prediction. The models resulting from this study can therefore account for more of the individual variability in body composition relating to how and where excess body fat is stored and how this body fat distribution changes with age, gender, and BMI.

The new prediction methods also likely show improved accuracy and clinical relevance over existing methods because of the inclusion of waist (for females) and hip (for males) circumferences in the final models. These measures have been previously established as predicting obesity status and risk of developing obesity–related health issues, such as type 2 diabetes, hypertension, insulin resistance, and dyslipidemia,^19^, ^20^, ^21^, ^22^ meaning that in addition to predicting body composition, these models may provide further insight into the development of more serious health risks.

Unlike the other referenced methods of determining body fat, the new regression model employs hand thickness as a predictor for body fat in men and women, with increased hand thickness correlating with decreased BFP. Previous research into hand muscle thickness, grip strength, and body composition has found that increased hand muscle thickness is correlated with increased skeletal muscle mass,^23^ while the relationship between grip strength and body composition has yielded mixed or inconclusive results.^24^, ^25^, ^26^ The inclusion of hand thickness in the final statistical model is likely significant because it can serve as a proxy for grip strength and whole‐body muscle mass and explain BFP when included alongside age and BMI.

While the models developed in this study account for overall body shape (from the waist, hip, and thigh circumferences) and localized body fat distribution (from the subscapular, abdominal, and thigh skinfolds measurements), they also account for the additional changes in body composition associated with age and BMI. The inclusion of age is especially important for accurate composition calculations because of the decrease in lean body mass and bone density that occurs with increasing age.^27^, ^28^, ^29^ Because the models include this variety of inputs, a trained clinician is likely required to collect the necessary measurements, whereas more simple methods like RFM^13^ can be determined by an individual without any clinical training. The extra requirements necessary for using these new models mean that they will likely be most useful in a clinical or medical setting where the ultimate goal is to most accurately determine BFP.

There were a few limitations for this study, mostly dependent on the population studied. While the study sample included a wide representation of age, race, and obesity levels, factors such as physical fitness and overall activity levels were not accounted for during recruitment. Because only working adults with full time jobs were eligible to participate in this study, the final prediction models are likely not applicable to special populations such as the elderly or athletes. The population studied was also limited to participants with a BMI of less than 40.0 kg m^−2^ because of inaccuracy of abdominal and thigh skinfold measures in morbidly obese individuals, so the results may not be applicable to working adults with extreme levels of obesity.

The findings of this study are clinically significant because they provide a method of accurately predicting BFP in American adults, without the need for any imaging or specialized body densitometry equipment. The only necessary equipment includes a skinfold caliper, tape measure, and anthropometer. With this equipment and proper training, it is much cheaper and easier to collect than imaging or densitometry methods. Some future directions may include developing methods only using a tape measure, in combination with age and BMI, so that individuals without any clinical training or equipment can collect the data required to accurately predict body composition.

## 5 CONFLICT OF INTEREST

The authors have no financial interests in relation to the work described in this research manuscript.
